# Change in Digital Cognitive Test Performance between Solanezumab and Placebo Groups in Preclinical Alzheimer’s Disease: Secondary Analyses from the A4 Study

**DOI:** 10.14283/jpad.2024.137

**Published:** 2024-07-24

**Authors:** Kathryn V. Papp, P. Maruff, D. M. Rentz, M. C. Donohue, A. Liu, P. S. Aisen, R. A. Sperling, A4 Study Team

**Affiliations:** 1 Department of Neurology, Brigham and Women’s Hospital and Harvard Medical School, Boston, Massachusetts USA; 2 Department of Neurology, Massachusetts General Hospital and Harvard Medical School, Boston, MA USA; 3 The Florey Institute of Neuroscience and Mental Health, University of Melbourne, Parkville, Victoria Australia; 4 Cogstate, Ltd, Melbourne, Victoria Australia; 5 Alzheimer Therapeutic Research Institute, Keck School of Medicine, University of Southern California, San Diego, CA USA; 6 60 Fenwood Road, Boston, MA 02115 USA

**Keywords:** Solanezumab, preclinical Alzheimer’s disease, clinical trial, digital cognitive assessment, computerized cognitive assessment

## Abstract

**Background:**

Primary results from the Anti-Amyloid in Asymptomatic Alzheimer’s disease Study (A4) suggested no benefit of solanezumab on its primary cognitive outcome, a composite of paper and pencil tests (the Preclinical Alzheimer’s Cognitive Composite; PACC).

**Objective:**

To determine whether change in cognitive performance, assessed using the Computerized Cognitive Composite (C3) summary score and C3 individual tests, differed between treatment groups over 240 weeks, differed based on baseline Aβ burden, and tracked with PACC decline.

**Design:**

Longitudinal analysis of cognitive change over 240 weeks on the C3 Summary Score and C3 individual tests between participants randomly assigned to solanezumab at a dose of up to 1600 mg intravenously every 4 weeks versus placebo.

**Setting:**

The A4 study took place at 67 sites in Australia, Canada, Japan and the United States.

**Participants:**

Cognitively unimpaired older adults (n=1117, Mean Age=71.9, 60.7% female) with elevated brain amyloid levels on 18F-florbetapir positron-emission tomography (PET) at baseline (n=549 in the solanezumab group; n=568 in the placebo group).

**Measurements:**

Participants completed the C3 battery and PACC every 6 months. The C3 Summary Score combines the Cogstate Brief Battery (CBB)-One Card Learning, the Behavioral Pattern Separation (BPS) Test- Object- Lure Discrimination Index, and the Face Name Associative Memory Exam (FNAME)- Face-Name Matching.

**Results:**

Change on the C3 Summary Score was moderately correlated with change on the PACC (Spearman’s corr=0.53, 95% CI: 0.49 to 0.57; p<0.001). At 240 weeks, mean change in the C3 Summary Score did not differ between groups; +0.24 in the solanezumab group and +0.27 in the placebo group (mean difference= −0.02; 95% CI: −0.13 to 0.08; p = 0.650). Lack of a treatment effect was similarly observed across most individual C3 tests. Performance on the C3 tests were influenced by level of amyloid burden, where higher levels were associated with worse performance.

**Conclusion:**

This study provides corroborating evidence that solanezumab does not slow cognitive decline in preclinical AD as exhibited with a computerized cognitive assessment with some evidence that solanezumab may exacerbate cognition on select digital outcomes. This study also provides important information that amyloid related cognitive change manifests differently on individual C3 tests.

## Introduction

In cognitively unimpaired (CU) older adults, abnormally high levels of amyloid (Aβ+) detected with neuroimaging or fluid biomarkers indicate that biological changes characteristic of Alzheimer’s disease (AD) have begun (1). Empirical and theoretical models estimate the time from this preclinical phase of AD to the clinical classification of dementia may be as long as 30 years (2), providing a substantial opportunity for interventions, such as those designed to interfere with accumulation of Aβ, to slow or prevent disease progression (3). By definition individuals with preclinical AD are cognitively unimpaired (CU), however prospective studies show that preclinical AD is characterised by insidious and progressive cognitive decline (4–6) which accompanies the continued accumulation of amyloid and tau (7), loss of brain volume (8), reduced brain activity (9) and faster progression to symptomatic disease (10). Consequently, cognitive decline is considered the main clinical symptom of preclinical AD and modification of its trajectory is considered a valid index of the efficacy of drugs designed to modify AD biology (11).

The Anti-Amyloid in Asymptomatic AD (A4) Study was the first clinical trial to evaluate the efficacy of a putative disease modifying drug, solanezumab, in sporadic preclinical AD: the monoclonal antibody that targets monomeric Aβ (12). The primary clinical outcome for A4 was the effect of the drug on cognitive decline, with the endpoint being a composite score of performance on standardized paper and pencil tests of memory, executive function and mental status termed the Preclinical Alzheimer Cognitive Composite (PACC) (13). The trial results confirmed that cognition declined subtly across the five-year study period in the A4 cohort but treatment with solanezumab did not alter the trajectory of cognitive decline (14). This finding halted development of solanezumab, however data arising from this study provide an important foundation for the design and interpretation of clinical outcomes for future disease-modifying therapies at the stage of preclinical AD.

The A4 study also provided an opportunity to determine the treatment effect of additional assessments of cognition other than the PACC. In the A4 study, a computerized cognitive test battery was used as a secondary outcome measure called the Computerized Cognitive Composite (C3) (15). The tests were selected based on their demonstrated sensitivity to cognitive decline in preclinical AD (15) and included the Behavioral Pattern Separation Test-Object version (BPSO) (16), the Face Name Associative Memory Exam (FNAME) (15, 17), and the Cogstate Brief Battery (CBB) (18, 19). In addition to the main performance scores from the individual tests in the C3, pre-study analyses identified performance measures that could be combined to provide a composite score with an optimal sensitivity to Aβ+ in CU adults. This was termed the C3 Summary Score (15). The aim of this study was to determine the effect of solanezumab on change in the C3 Summary Score, and on the individual tests in the C3 battery. Second, the extent to which Aβ burden at trial entry influenced the rate of decline on the C3 across the 240-week trial period was explored. Finally, the extent to which cognitive change measured by the C3 summary score was associated with the PACC was examined.

## Methods

### Participants and Study Design

The A4 Study design and aims have been described in detail previously (14). Briefly, A4 was a double-blind, placebo-controlled 240-week Phase 3 trial of an anti-Aβ monoclonal antibody in CU older adults with preclinical AD (12) occurring across 67 sites. Participants interested in enrolling in A4 were required to be aged 65 to 85 and were deemed CU based on Mini Mental Status Exam (MMSE) ranging from 25–30 and Global Clinical Dementia (CDR) Rating Score of 0. They were also required to be classified as Aβ+ based on results from a florbetapir Positron Emission Tomography (PET) scan.

### Amyloid PET Imaging

Eligible participants completed a florbetapir PET scan prior to enrollment. Scan acquisition occurred over 50–70 minutes following an injection of 10mCi of florbetapir-F18. Aβ binding was assessed using mean standardized uptake value ratio (SUVr) with whole cerebellar gray as a reference region. Participants were deemed eligible (Aβ+) versus not eligible (Aβ−) using an algorithm combining both quantitative SUVr (>1.15) information and a centrally-determined visual read (12). An SUVR of 1.10 to <1.15 was considered elevated only when a visual read was considered positive by a two-reader consensus. SUVR’s were converted to centiloids expressing amyloid level on a scale of 0 to 100.

### Treatment

Participants were randomly assigned to receive either placebo or intravenous solanezumab. The dose was initially 400 mg intravenously every 4 weeks, but as previously described (14), the protocol was changed to adjust the dose to 1600 mg intravenously every 4 weeks in 2017, when approximately 970 participants had already been enrolled. Concurrently, the double-blind phase of the trial was extended to 240 weeks.

### Assessments

#### Digital Cognitive Tests: The C3 Battery

The C3 battery is a supervised and administrator-guided computer based cognitive assessment that has been described in detail (15, 20). For the A4 study the C3 battery was presented to all participants using an Apple iPad. The tests included in the C3 battery are described below.

Behavioral Pattern Separation- Object (BPS-O; recently renamed the Mnemonic Similarity Test): Participants are serially presented with images of 40 everyday objects and are allotted 5 seconds to determine whether the item is for use “indoors” or “outdoors” to ensure adequate attentiveness to stimuli (16). Participants are subsequently shown 20 of the same items interspersed with both novel images and lure images. They are asked to categorize each image as: Old, Similar, or New within 5 seconds. Accuracy and Reaction Time (RT) measures are collected. Of interest is the rate at which participants can correctly identify lures as “Similar” rather than as “Old.” The lure discrimination index (LDI) is computed as the proportion of “Similar” responses given to lure items minus the ratio of “Similar” responses given to the foils (the latter is to correct for response bias). The LDI is the primary outcome from the BPS-O task. A higher LDI indicates better pattern separation performance (16, 21).

Face-Name Associative Memory Exam (FNAME): Participants are shown, one at a time, 12 face-name pairs (15, 17). For each face-name pair, the participant is asked whether the name “fits” or “doesn’t fit” the face to ensure adequate attentiveness to the stimuli. Participants are allowed 5 seconds to respond and are asked to try to remember the face-name pair. After the learning phase there was a filled delay of 12 to 15-minutes comprised of the CBB. After the delay, three types of memory are elicited: a face recognition trial (FSBT), first letter name recall (FNLT) and face-name matching (FNMT). For the FSBT, participants are asked to identify the previously learned faces, presented alongside two distractor faces of matching age, race, and sex. The target face is subsequently presented with a touchscreen keyboard and the participant selects the first letter of the name paired with that face (FNLT). Finally, the target face is presented with three names (target name, a re-paired same-sex name, and an age and sex-matched foil name) and the participant must select the correct name (FNMT). Accuracy for each component is scored /12 with FNMT number of correct matches serving as the primary outcome.

Cogstate Brief Battery (CBB): The CBB (18, 19) uses playing cards as stimuli and includes a measure of psychomotor function (Detection, DET), attention (Identification, IDN), working memory (One-Back Test, ONB), and visual memory (One-Card Learning, OCL). Measures of average reaction time (RT) of correct responses (milliseconds) and accuracy (ACC, proportion correct) are recorded for each test. To normalise distributions of performance data, a log10 transformation was applied to RT measures and an arcsine square root transformation applied to ACC measures. For each test in the CBB, participants are shown a single facedown card in the centre of the display, which flips at variable intervals to reveal the face card. With this flip, the participant must respond as fast and as accurately as possible by pressing manually a “yes” or “no” button. For DET, participants are required to press the yes key in response to the card turning face-up. The task continues until 35 correct trials are recorded. For IDN, participants must press the yes or no key in response to the question “Is the card red”. Thirty correct trials are required. For OCL, participants must press the yes or no key in response to the question “Have you seen this card before in this test?”. For this test, four cards drawn at random from the deck, are shown repeatedly interspersed with other non-repeating cards in a pseudo-random order. Eighty trials are given on which 36 responses require a yes answer. For OBK, participants must press the yes or no key in response to the question “Does this card match the card as shown on the previous trial?”. Thirty-five trials are given. Performance measures included speed (log10RT, e.g., RT) for DET, IDN and OBK and accuracy (arcsine proportion correct, e.g., ACC) for IDN, OBK and OCL.

#### The C3 Summary Score

The C3 Summary Score, which has been described previously (15), combines three memory-based scores from the digital tests: (1) the BPSO lure discrimination index, (2) the FNAME accuracy from the face-name matching task (FNMT), and (3) accuracy of performance on the OCL from the CBB. The C3 Summary Score was created in part to maximize the signal to noise ratio in adults with preclinical AD when compared to a well-matched group whose amyloid levels had remained within normal limits. For the C3 Summary Score, a decreasing value indicated that cognition was worsening over time and vice versa. To provide a common context for interpreting performance on the C3 battery from the individual computerized tests, timed performance measures (DET-RT, IDN-RT, IDN-RT, ONB-RT) were reverse coded to indicate a negative value for a worse performance.

### Statistical Analyses

All analyses were conducted using R (R-project.org). Demographic differences at baseline between placebo versus solanezumab groups were assessed using Welch’s two-sample t-tests for continuous variables and Fisher’s Exact test for categorical variables (e.g., age, APOE).

#### Effect of solanezumab on cognition measured with the C3 battery

The statistical method applied to determine the effect of solanezumab on the C3 Summary Score, and on the performance measures for the individual computerized tests was identical to that applied in the primary statistical analyses for A4 (14). Specifically, for each outcome, a natural cubic spline model was used to assess the difference between treatment groups in change from baseline to 240 weeks, assuming a heterogeneous Toeplitz variance-covariance. The dependent variable in these analyses was the score at baseline and at each post-baseline visit. Time in the study was treated as a continuous variable with values equal to the years between baseline and follow-up exam dates. The spline basis expansion assumed an interior knot at the median of observation times, and boundary knots at zero years and the maximum follow-up (23). Fixed effects in the model included the following terms: (i) spline basis expansion terms (two terms), (ii) spline basis expansion terms-by treatment interaction (two terms), (iii) test version administered (where applicable), (iv) age, (v) education, (vi) APOE4 Carrier Status (yes/no), and (vii) baseline florbetapir cortical SUVr. The model was constrained to not allow any difference between treatment group means at the baseline assessment.

#### Quantifying cognitive change using performance on tests in the C3 battery

The magnitude of change on the C3 Summary Score, and the performance scores from the individual tests in the C3 battery over the course of the trial was examined using a standardized scale to allow comparison between outcomes. Modelled mean and 95% confidence intervals (CI) for continuous outcomes (divided by the baseline standard deviation) were derived for the final assessment in the trial (week 240) from the spline models. Estimates from binominal outcomes were estimated by longitudinal logistic regression models fit by generalized estimating equations. For the week 240 assessment, modelled mean standardized change in accuracy (on the scale 0 to 1 divided by the baseline standard deviation) from baseline, and mean group differences in change in accuracy were marginal estimates derived by average subject-specific estimates. The 95% confidence intervals (95%CIs) for these marginal estimates were derived by bootstrap resampling of participants with replacement 500 times.

#### Influence of amyloid burden at trial entry on cognitive decline

The pre-specified statistical analysis indicated that there was no effect of solanezumab on the C3 Summary Score. We therefore explored the extent to which change over time on the C3 summary score, and individual test scores was influenced by initial Aβ burden at baseline. To achieve this, each participant’s baseline Aβ level was classified into one of three categories by centiloid (CL) tertiles: <46.1, 46.1 to 77.2, and >77.2 CL. Performance data for the C3 battery tests were then collapsed across treatment conditions, and modelled means for change from baseline at the week-240 visit were computed for the C3 summary score, and for the individual tests, by adding baseline Aβ tertile to, and removing the treatment term from, the statistical model. For the C3 summary score, rates of change across all visits were plotted separately for each Aβ tertile group. Additionally, group mean standardized change from baseline scores, and their 95% confidence intervals, at the week 240 assessment were computed for each Aβ tertile using the method described for treatment group means. Because this analysis was exploratory, formal statistical comparisons were not made between Aβ tertiles for any cognitive score. Instead, group mean change from baseline scores for cognitive test scores in different baseline amyloid level tertiles was inferred to be different if there was no overlap between the 95%CIs associated with the relevant group means. Similarly, where test performance was characterized by an improvement or decline from baseline, the improvement or decline was classified as true if the confidence intervals for a group mean did not include zero.

#### Associations between cognitive decline measured using the PACC and C3 Summary Score

The association between the C3 summary score and the PACC at the baseline and week 240 visit was examined using Spearman’s rank correlation at the baseline and week 240 visit. Strength of association between the C3 Summary Score and PACC scores were also computed between change from baseline to week 240 scores. The change from baseline scores to week 240 scores for the C3 Summary Score and PACC score were derived from spline models with participant-specific random intercepts and slopes.

## Results

### Participants

There were no differences at baseline between participants who received placebo or solanezumab on demographic characteristics such as age, sex, education, race, or ethnicity. Additionally, there were no differences between groups on clinical characteristics such as MMSE score, mean florbetapir SUVr, or in the proportion of APOE ε4 carriers (Table 1).

**Table 1:** Baseline Participant Characteristics by Treatment Group

	**Placebo n=568**	**Solanezumab n=549**	**Total n=1117**	**p-value**
Age M(SD)	71.9 (5.0)	71.9 (4.6)	71.9 (4.8)	0.990
Sex (% female)	60.7%	59.4%	60.1%	0.643
Education M(SD)	16.6 (2.9)	16.6 (2.7)	16.6 (2.8)	0.763
MMSE	28.8 (1.3)	28.8 (1.3)	28.8 (1.3)	0.538
Race				0.694
American Indian or Alaskan Native	0 (0.0%)	1 (0.2%)	1 (0.1%)	
Asian	5 (0.9%)	2 (0.4%)	7 (0.6%)	
Black or African American	15 (2.6%)	12 (2.2%)	27 (2.4%)	
More than one race	3 (0.5%)	5 (0.9%)	8 (0.7%)	
Unknown or not reported	3 (0.5%)	3 (0.5%)	6 (0.5%)	
White	542 (95.4%)	526 (95.8%)	1068 (95.6%)	
Ethnicity				0.910
Hispanic or Latino	18 (3.2%)	16 (2.9%)	34 (3.0%)	
Not Hispanic or Latino	545 (96.0%)	527 (96.0%)	1072 (96.0%)	
Unknown or unreported	5 (0.9%)	6 (1.1%)	11 (1.0%)	
Florbetapir SUVr	1.3 (0.2)	1.3 (0.2)	1.3 (0.2)	0.996
APOE Genotype (% ε4+)	335 (59.0%)	327 (59.6%)	662 (59.3%)	0.843

NOTE. Two-sample t-test with unequal variances were used for continuous variables and Fisher’s Exact test for categorical variables.

### Missing Data

Missing data for the C3 battery data increased across progressive study visits. For example, 8.9–9.3% of administrations were missing at week 60, 12.6–14.8% at week 108, 20.6–20.8% at week 156, and 28.9–29.7% at week 228. However, rates of data missingness did not differ between treatment groups and were consistent with rates of participant attrition described previously for the A4 study (14).

### Effect of solanezumab on cognition measured with the C3 battery

**Figure 1:**
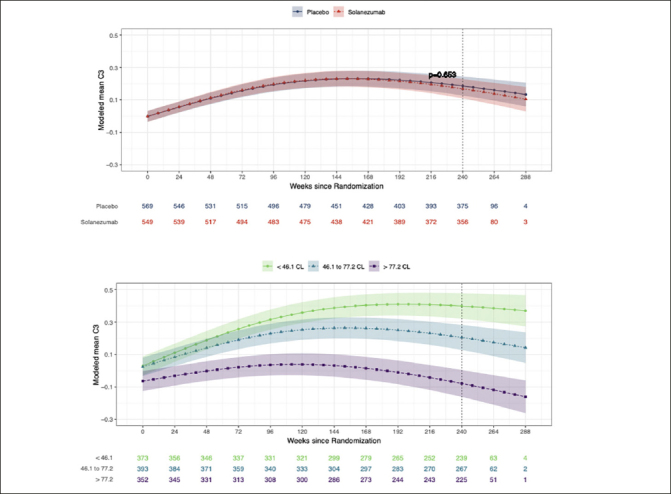
The effect of solanezumab on cognitive performance: The C3 Summary Score modeled over the course of the trial by treatment group and by baseline amyloid level Modeled mean C3 summary scores over time by treatment group (Fig 1a) and by tertiles of baseline amyloid PET (Fig1b). Each model assumes a natural cubic spline for time with two degrees of freedom per group and controls for age, APOEe4 carriage, and education; and further assume a heterogeneous unstructured covariance structure. The model for treatment group (top panel) constrains the group means to be the same at baseline, while the model on the bottom panel relaxes this constraint. Shaded regions are 95% confidence intervals. For both figures lower scores over time represent a decline from baseline in cognition. For Fig 1a and 1b, values given below each x-axis visit represent the sample size remaining in the study at each visit week from randomization. The vertical dotted line represents the final visit in the trial (Week 240). CL = centiloid.

Figure 1a summarizes modelled change from baseline scores across the study period for the C3 Summary Score in the solanezumab and placebo groups. The treatment effect estimated from the omnibus statistical model did not reach the criterion for statistical significance (Figure 1). Table 2 shows the modelled standardized group mean change from baseline to 240 weeks for the C3 summary score under placebo and solanezumab as well as the difference between these change from baseline means. Table 2 also shows how the difference in the change from baseline means between the treatment and placebo groups were not significantly different. Figure 1b shows the results of analyses comparing change over time on the C3 summary score between participants with increasing baseline amyloid levels (averaged over treatment condition). Figure 1b shows clear separation in change over time between the baseline amyloid level groups. Groups with higher baseline amyloid level showed worse performance at the week-240 visit than those with lower baseline amyloid levels (i.e. no overlap between 95%CIs associated with each spline fit).

**Table 2:** Standardized change (by baseline standard deviation) on the PACC, C3 Summary Score and individual measures from the C3 battery by treatment group

	**Metric**	**Modeled mean standardized change Placebo**	**Modeled mean standardized change Solanezumab.**	**Modeled mean standardized difference vs. placebo (95% CI)**	**p value**
PACC	Standard scores	−0.42	−0.53	−0.11 (−0.30 to 0.08)	0.260
C3 Summary Score	Standard scores	0.27	0.24	−0.02 (−0.13 to 0.08)	0.669
Performance on individual tests					
BPS-O Lure Discrimination Index (LDI)	Discrimination index	0.20	0.24	0.03 (−0.06 to 0.13)	0.502
FNAME 1st Letter Name Recall (FNLT)	Accuracy	−0.23	−0.17	0.06 (−0.02 to 0.14)	0.205
FNAME Face-Name Matching (FNMT)	Accuracy	0.12	0.09	−0.03 (−0.12 to 0.06)	0.558
FNAME Face Recognition (FSBT)	Accuracy	−0.10	−0.23	−0.13 (−0.24 to −0.02)	0.030
CBB Detection (DET-RT)	Speed	−0.10	−0.15	−0.04 (0.07 to −0.16)	0.479
CBB Identification (IDN-RT)	Speed	−0.09	−0.09	−0.01 (0.10 to −0.11)	0.886
CBB Identification (IDN-ACC	Accuracy	0.04	−0.06	−0.10 (−0.30 to 0.09)	0.287
CBB One-Back Test (ONB-RT)	Speed	0.06	0.03	−0.02 (0.07 to −0.12)	0.600
CBB One-Back Test (ONB-ACC)	Accuracy	−0.02	−0.27	−0.25 (−0.42 to −0.05)	0.005
CBB One Card Learning (OCL-ACC)	Accuracy	0.24	0.22	−0.01 (−0.12 to 0.08)	0.794

Note: FNAME=Face Name Associative Memory Exam, CBB=Cogstate Brief Battery (CBB)

### Quantifying cognitive change using performance on individual C3 battery

**Figure 2:**
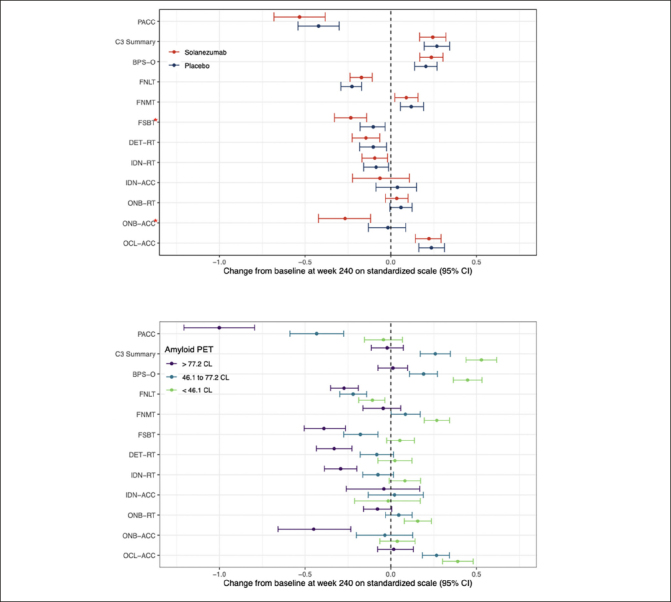
The effect of solanezumab on cognitive performance: Modeled 240 Week Change and 95% CI for individual tests form the C3 battery Change at 240 weeks by treatment group (Fig 2a) and by tertiles of baseline amyloid PET (Fig 2b). Each model assumes a natural cubic spline for time with two degrees of freedom per group and control for age, APOEe4 carriage, and education; and further assume a heterogeneous unstructured covariance structure. The model for treatment group (top panel) constrains the group means to be the same at baseline, while the model on the bottom panel relaxes this constraint. Shaded regions are 95% confidence intervals. Nominally significant (nominal p<0.05) treatment effects were seen with FSBT and ONB-Acc (red asterisks). Reaction time (RT) outcomes have been inverted so that low scores indicate worse performance across all outcomes. BPS-O=Behavioral Pattern Separation-Object Lure Discrimination Index, FNLT=Face Name Associative Memory Exam (FNAME) 1st letter name recall, FNMT=FNAME Face-Name Matching, FSBT=FNAME Face Recognition, DET-RT=Cogstate Brief Battery (CBB) Detection Reaction Time, IDN-RT=CBB Identification Reaction Time, IDN-ACC =C BB Identification Accuracy, ONB-RT=CBB One-Back Reaction Time, ONB-ACC = One-Back Accuracy, OCL-ACC=CBB One-Card Learning Accuracy.

Figure 2a summarizes the mean standardized change from baseline to the week-240 for the C3 summary score and performance on the individual C3 tests for the solanezumab and placebo treatment groups. The 95%CI is also shown for each standardized group mean change score. The group mean standardized change from baseline score for the PACC is also shown on Fig 2a for context. Fig 2b summarizes the group mean standardized change from baseline scores (and their associated 95%CIs) for the same cognitive outcomes in the entire study sample classified according to baseline Aβ level, expressed in tertiles. For both Fig 2a and 2b, negative mean group standardized change from baseline indicates cognitive decline. The magnitude and statistical significance of the difference between the solanezumab and placebo treatment group mean change from baseline scores is summarised on Table 2 for each C3 test outcome. Fig 2a shows that the C3 summary score improved from baseline to the same extent for the placebo and solanezumab treatment groups (i.e., 95%CIs do not include 0). By comparison, performance on the PACC declined from baseline in the placebo and solanezumab treatment groups and to the same extent (i.e., 95%CIs do not include 0). The results of the statistical analyses summarised on Table 2 confirm that differences in change from baseline between treatment conditions were not statistically significant for the C3 summary score or for the PACC.

Inspection of standardized change from baseline for the performance scores from the individual C3 tests, summarised on Fig 2a, indicates that performance declined across the study period with an equivalent magnitude for the FSBT, DET-RT and IDN-RT outcomes (i.e., 95%CIs do not include 0). No decline from baseline to 240 weeks was observed for the IDN-ACC, ONB-RT outcomes (i.e., 95CIs included 0). For the ONB-ACC and FSBT outcomes, decline from baseline in the solanezumab treatment group was greater than any decline from baseline in the placebo group (Fig 2a) and for both tests this difference was sufficient to reach statistical significance (Table 2). For each outcome measure from the C3 battery, the magnitude of decline from baseline, or absence of improvement from baseline, at 240 weeks was not as large as the decline from baseline observed for the PACC (Figure 2a).

Figure 2b shows the changes from baseline at the week-240 visit for the C3 summary score for groups with increasing baseline amyloid levels that was shown on Fig 1b. Considering the 95%CIs associated with mean standardised change scores in each baseline amyloid level group, the data in Fig 2b show for the <46CL group, performance improved substantially from baseline, while for 46.1 to 77.2 CL group performance also improved but to a lesser extent. For the >77.2CL group, performance did not change from baseline. This relationship between baseline amyloid level and magnitude of change in cognition was qualitatively similar and mirrored that observed for the PACC, where the magnitude of decline from baseline became greater as baseline amyloid level increased. The effect of baseline amyloid burden on cognitive change was classified as occurring if there was no overlap between the upper (95%ile) CI bound associated with mean standardized change from baseline in the <46.1CL group and the lower (5%ile) CI bound associated with group mean standardized change from baseline in >77.2CL group. Accordingly, baseline amyloid level related change was observed for the BPS-O, FNMT, FSBT, DET-RT, IDN-RT, ONB-RT, ONB-ACC, and OCL-ACC outcomes (Fig 2b). Of these, increasing baseline amyloid levels manifest as greater decline for the FNLT, DET-RT, IDN-RT, FSBT and ONB-ACC outcomes. The effect of baseline amyloid on cognition manifest as a reduced improvement for the BPS-O, FNMT, OCL-ACC and ONB-ACC outcomes. Baseline amyloid level did not influence change in performance on the IDN-ACC score.

### Associations between cognitive decline measured using the PACC and C3 Summary Score

**Figure 3:**
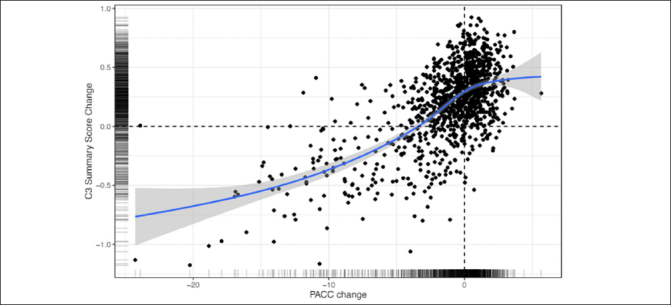
Correlation between Change on the PACC and Change on the C3 Summary Score over 240 weeks across both Solanezumab and placebo groups C3= Computerized Cognitive Composite; PACC=Preclinical Alzheimer’s Cognitive Composite

Figure 3 shows the association between change from baseline to 240 weeks for the PACC and the C3 Summary Score. The association was statistically significant and moderate in magnitude (Spearman’s r = 0.73 (95% CI 0.70–0.76)). Computation of associations between the C3 Summary Score and PACC were slightly weaker at baseline (Spearman’s r = 0.62 (95% CI 0.58–0.65).

## Discussion

The results of this study investigating the effect of solanezumab on cognition in preclinical AD, as measured by the C3 battery, indicated there was no difference in the trajectory of cognitive change across 240 weeks between the placebo and solanezumab treated groups (Fig 1a). The results from the C3 summary score support the A4 analyses that treatment with solanezumab did not influence the rate of decline on the PACC (14) and that solanezumab had no benefit on cognition in preclinical AD.

The A4 study was the first to test the hypothesis that disrupting the accumulation of amyloid would prevent worsening of symptoms in sporadic preclinical AD, where symptoms were defined by the nature and severity of cognitive decline. While treatment with solanezumab did reduce accumulation of amyloid in the A4 study (14) there was no evidence of any treatment benefit to cognition in that initial study or in this study. However, two recent and independent studies conducted in older adults with early symptomatic AD, using the monoclonal antibodies, lecanemab and donanemab, did observe large drug-related reductions in amyloid levels following treatment with these drugs. Furthermore, for both drugs, lowering amyloid levels resulted in a reduction in the trajectory of cognitive decline (27, 28). The positive effects of these drugs in symptomatic AD suggests the absence of a cognitive benefit from solanezumab occurred because solanezumab may not have reduced amyloid levels sufficiently, rather than because the amyloid reduction did not influence cognition per se. The ability of both lecanemab and donanemab to reduce amyloid and improve cognition is now being explored in preclinical AD, with some of these clinical trials using the same cognitive outcomes as those used in the A4 study (NCT04468659, NCT06384573, NCT05026866). Thus, despite the absence of a drug effect on cognition, cognitive data from the A4 study will provide an important context for interpreting outcomes from those trials.

### Reduced improvement in performance on C3 summary score in preclinical AD

One key difference in the effect of solanezumab on PACC and on the C3 summary score was that both the placebo and solanezumab groups showed a decline from baseline to the week 240 visit of approximately 0.5 standard deviation units on the PACC (Fig 2a). However, for the C3 summary score, change in performance over the same period was characterised by an improvement of approximately 0.25 standard deviation units for both the solanezumab and placebo groups (Fig 1a, Table 2). This improvement was unexpected given that each memory test contributing to the C3 summary was designed to use alternate versions to minimize practice effects associated with repeated content. The general improvement in performance on the C3 summary score could reflect this score had a low sensitivity to the cognitive decline that characterizes preclinical AD. Contrary to this hypothesis, the data summarized on Fig 2b show that standardized change from baseline at the week 240 visit, on both the C3 and PACC scores, was influenced by baseline amyloid levels. For the PACC, higher baseline amyloid levels led to greater cognitive decline. For the C3 summary score, higher baseline amyloid levels were associated with a reduced improvement in performance over the same period (Fig 2b). The consistency of these effects is also evident in the moderate associations observed on the C3 summary score and PACC at baseline and week 240, as well as change from baseline to the week 240 visit. The data suggests that, like the PACC, the C3 summary score was sensitive to amyloid levels in CU older adults. Improvement in the C3 summary score most likely resulted from the many repeated assessments given in the A4 study, although this improvement was reduced in adults with higher baseline levels of amyloid (Fig 1b, Fig 2b).

### Effect of solanezumab on individual C3 tests

In addition to the C3 Summary Score, the effect of solanezumab was examined for the individual tests in the C3 battery. The post-hoc and non-corrected nature of these analyses means that generalizations about any effects must be interpreted with caution. Nonetheless, the nature and magnitude of change from baseline to the week 240 visit on the individual C3 tests suggests three main types of performance (Fig 2a). First, there were tests for which performance declined from baseline in the placebo and solanezumab groups. These tests measured the efficiency of delayed “free” recall on the face name test (FNLT) along with the speed of performance on tests of psychomotor function and attention from the CBB (DET-RT, IDN-RT). Second, there were tests for which no change from baseline was observed in both placebo and solanezumab groups, including accuracy of performance on the CBB attentional test (IDN-ACC), and speed of performance on the CBB working memory test (ONB-RT). Third, there were tests for which performance improved from baseline in both placebo and solanezumab groups. These included the accuracy of performance on the tests of pattern separation processes in memory (BPS-O, OCL-ACC) and multiple-choice delayed recall on the face name test (FNMT). While the speed of simple attentional processing declined over time in preclinical AD, repeated exposure to the computerized tests that measured demanding episodic memory processes such as pattern separation improved with repeated testing (Fig 2a).

In preclinical AD, decline in attentional function and delayed recall efficiency could reflect a general effect of aging in these adults over the 4 years of the study (4–6). However recent studies show an early site of amyloid-related tau accumulation is in the locus coeruleus (32), a brain region important for arousal and vigilance (33). Thus, the loss of these attentional and memory efficiency functions may be an AD-related cognitive effect. This will be resolved by studying these same aspects of cognition over the same period in matched adults who do not have abnormal levels of amyloid. In contrast, improvement in performance on the tests of episodic memory (OCL-ACC, BPS-O, FNMT) that were selected as part of the C3 summary score, originally based on their sensitivity to amyloid-related cognitive decline (34–36), improved over the A4 study period. This systematic improvement likely reflects practice effects that are commonly observed on memory tests in natural history studies (29, 30) despite the fact that the memory tests on the C3 were designed to have alternate forms. The performance improvement observed in A4 may be a consequence of treatment expectations (i.e., a placebo effect) (37) or, arise from the more frequent reassessments in the A4 study, allowing participants to habituate to the task, compared to the natural history studies from which they were selected (e.g., every 6 months versus annually). In a trial of solanezumab in the dominantly-inherited AD prevention trial, the placebo group did not exhibit as much decline as was expected based on modelling from observational data (38). It is worth noting that performance improvement over time on the C3 was not observed for all memory measures of episodic memory. For example, performance on tests that assessed free recall efficiency (FNLT) and recognition memory (FSBT) showed a decline across the solanezumab and placebo groups. Future studies may wish to select the tests that are sensitive to decline rather than those that stay stable or improve.

### Concordance between C3 change and PACC change

Finally, the results indicated that decline on the C3 summary score was moderately associated with decline on the PACC (Fig 3). This relationship suggests that computerized measures may be used in future trials of disease modifying therapies conducted in preclinical AD. There are several advantages of computerized cognitive assessments versus traditional paper and pencil measures including more automated administration and scoring and more precise measurement (e.g., reaction time). While most clinical trials rely on traditional paper and pencil cognitive measures as primary outcomes, there is increasing interest in computerized cognitive assessments, particularly with the rise of decentralized and largely remote clinical trial designs (39). The moderate correlation observed between PACC change and C3 change observed in this trial suggests that computerized measures may capture concordant but also potentially unique information about cognitive change, providing support for their inclusion as outcomes in future trials.

## Limitations and Conclusion

In addition to those noted above, there are several other broader limitations worth mentioning. First, as this was a clinical trial sample it is limited in the extent to which it represents all adults at risk for AD, especially given the low proportion of participants from minority and culturally diverse groups. Additionally, assessments in the A4 trial were complicated by a midtrial dose increase (14) as well as by the disruption to clinical care caused by the COVID-19 pandemic. Despite these limitations, the extent to which amyloid influenced performance on the tests in C3 battery was consistent with those observed on the paper and pencil primary outcome and supports the use of computerized measures in clinical trials. This study also provides important information that the pre-specified C3 summary score derived from the A4 screening data may not be the most sensitive for detecting amyloid-related change over time. Future work will explore the individual tests identified as changing in this study at various amyloid levels to determine if there is a more optimal C3 summary score of change over time. Finally, while the A4 study was unsuccessful in demonstrating any cognitive benefit from solanezumab, the data from this large and well-conducted trial provides important information for the design of future studies and for the interpretation of their results.

## Notes

*Ethical Standards:* Approval from an institutional review board was obtained at each of the sites and all participants and their study partners provided written informed consent prior to data collection.
